# Identification of oncofetal PIWI-interacting RNAs as potential prognostic biomarkers in non-small cell lung cancer

**DOI:** 10.3389/fgene.2025.1611805

**Published:** 2025-08-29

**Authors:** Michelle E. Pewarchuk, Vanessa G. P. Souza, David E. Cohn, Nikita Telkar, Greg L. Stewart, Katya H. Bénard, Patricia P. Reis, Victor D. Martinez, Wendy P. Robinson, Wan L. Lam

**Affiliations:** ^1^ British Columbia Cancer Research Institute, Vancouver, BC, Canada; ^2^ Interdisciplinary Oncology Program, University of British Columbia, Vancouver, BC, Canada; ^3^ Department of Medical Genetics, University of British Columbia, Vancouver, BC, Canada; ^4^ British Columbia Children’s Hospital Research Institute, Vancouver, BC, Canada; ^5^ Department of Surgery and Orthopedics, Faculty of Medicine, São Paulo State University (UNESP), Botucatu, São Paulo, Brazil; ^6^ Department of Pathology and Laboratory Medicine, IWK Health Centre, Halifax, NS, Canada; ^7^ Department of Pathology, Dalhousie University, Halifax, NS, Canada

**Keywords:** lung cancer, PIWI-interacting RNAs, oncofetal biomarkers, lung adenocarcinoma, lung squamous cell carcinoma, non-coding RNAs

## Abstract

Lung cancer is the leading cause of cancer-related deaths worldwide, with non-small cell lung cancer (NSCLC) accounting for the majority of these cases. Despite advancements in targeted therapies, early detection remains a significant challenge, highlighting the need for novel biomarkers. This study investigates the role of PIWI-interacting RNAs (piRNAs) in lung cancer, specifically focusing on their potential as oncofetal biomarkers in lung adenocarcinoma (LUAD) and lung squamous cell carcinoma (LUSC), the two most common histological subtypes of NSCLC. We hypothesize that piRNAs exhibit oncofetal expression patterns and may contribute to lung cancer development. Through bioinformatics analysis, we identified distinct piRNA profiles in non-neoplastic, malignant, and fetal lung tissues. Among these, 37 piRNAs in LUAD and 46 piRNAs in LUSC displayed oncofetal expression, meaning they were present in tumor tissues but absent in adjacent normal lung tissue. These oncofetal piRNAs showed significant prognostic value in both LUAD and LUSC cohorts, with a specific signature of eight oncofetal piRNAs predicting high-risk patients in LUAD. We validated the robustness of this signature in a separate in-house cohort, which underscores its potential as a prognostic biomarker. Our findings suggest that oncofetal piRNAs could offer new diagnostic and therapeutic opportunities, particularly for early detection.

## 1 Introduction

Lung cancer remains one of the most commonly diagnosed cancers and the leading cause of cancer-related mortality worldwide, making it a priority for research aimed at identifying novel therapeutic targets and biomarkers (Siegel et al., 2024). Lung cancer is broadly classified into two main histological types: small cell lung cancer (SCLC) and non-small cell lung cancer (NSCLC), with NSCLC accounting for approximately 85% of cases. NSCLC is further divided into adenocarcinoma (LUAD), squamous cell carcinoma (LUSC), and large cell carcinoma (LCC), with LUAD and LUSC being the predominant subtypes (Herbst et al., 2018). These subtypes differ in their cell of origin: LUAD typically arises from distal glandular cells in the alveoli, while LUSC originates from the proximal airway epithelium (Cheung and Nguyen, 2015). Molecular advances have identified key, targetable genomic alterations in NSCLC (particularly LUAD), including *EGFR, BRAF,* and *KRAS* mutations, and *ALK* translocations, leading to the development of targeted therapies as well as diagnostic markers (Cancer Genome Atlas Research Network, 2014; Chen et al., 2014; Du et al., 2018; Herbst et al., 2018; Tan and Tan, 2022). However, lung cancer is diagnosed predominantly at advanced stages and these treatments are prone to resistance, resulting in poor survival outcomes. Therefore, strengthening our understanding of the molecular pathogenesis of lung cancer and identifying novel molecules involved in tumorigenesis can drive the development of effective treatments and biomarkers, ultimately improving patient prognosis (Herbst et al., 2018; Cassim et al., 2019).

Increasing evidence supports that oncofetal reprogramming, where tumor cells co-opt signaling pathways typically active only during embryonic development, has significant implications for cancer biology and potential therapeutic strategies (Sharma et al., 2022; Cao et al., 2023). Oncofetal reprogramming has been observed in various cancers where gene expression patterns resembling early developmental stages of the corresponding organ have been identified in tumor profiles (Coulouarn et al., 2005; Huang et al., 2019; Hassan et al., 2009; Feng et al., 2014; Hu and Shivdasani, 2005). Some oncofetal molecules have entered clinical application as early diagnostic markers, therapeutic targets, or prognostic indicators for various tumors. For example, alpha-fetoprotein (AFP) and carcinoembryonic antigen (CEA) serve as diagnostic biomarkers for hepatocellular carcinoma (Mizejewski, 2002; Chen et al., 2020), and colorectal cancer (Das et al., 2017; Feng et al., 2018), respectively.

Small non-coding RNAs (sncRNAs) represent a diverse class of RNA molecules that, despite lacking protein-coding potential, play crucial roles in gene regulation (Fu, 2014). They include microRNAs (miRNAs), transfer RNA-derived fragments (tRFs), small nucleolar RNAs (snoRNAs), small nuclear RNAs (snRNAs), small interfering RNAs (siRNAs), and PIWI-interacting RNAs (piRNAs) (Fu, 2014). Our group previously demonstrated that miRNAs exhibit oncofetal expression patterns in lung cancer (Cohn et al., 2021). Furthermore, we showed that oncofetal miRNAs regulate NFIB, a transcription factor essential for lung differentiation and maturation during development (Becker-Santos et al., 2016).

While miRNAs have been extensively studied, piRNAs remain largely unexplored in lung cancer, despite their abundance, evolutionary conservation, and established roles in gene regulation and development (Mei et al., 2013). Initially characterized for their function in transposon regulation and germline development (Halic and Moazed, 2009; Iwasaki et al., 2015; Teixeira et al., 2017), piRNAs have shown expression in multiple somatic tissues and cancers, where they may contribute to tumorigenesis. A recent pan-cancer analysis revealed widespread piRNA expression across normal and malignant tissues from 11 organ types, with distinct tissue-specific profiles, particularly in the thyroid and prostate (Martinez et al., 2015). Certain piRNAs have been associated with patient survival, suggesting their potential as cancer biomarkers (Martinez et al., 2015). However, their functional significance in lung cancer remains largely unknown, and few piRNAs have been characterized to date, highlighting the need for further investigation.

We sought to explore whether other ncRNA classes, such as piRNAs, might also exhibit oncofetal characteristics and contribute to lung cancer progression. In this study, we characterized the piRNA landscape in LUAD and LUSC, focusing on their potential role as oncofetal markers. Like miRNAs, we hypothesize that specific piRNAs may display oncofetal expression patterns, potentially reflecting their involvement in cancer development. We utilized a bioinformatics framework previously applied to miRNA discovery to identify and classify piRNAs based on their expression patterns in non-neoplastic, malignant, and fetal lung tissues. Furthermore, we evaluated the association of oncofetal piRNAs with patient survival and established an oncofetal piRNA prognostic signature for LUAD.

## 2 Methods

### 2.1 Data collection

This study utilized four cohorts: The Cancer Genome Atlas (TCGA) cohort, which included paired tumor and adjacent adult non-neoplastic lung tissue (ANL) samples for LUAD and LUSC; a fetal lung (FL) cohort comprising human fetal lung samples; and a validation cohort from the British Columbia Cancer Agency (BCCA) containing LUAD and paired ANL tissue samples. Small RNA sequencing (small RNA-seq) data for paired samples (tumor and ANL) in the TCGA LUAD (n = 46 pairs) and TCGA LUSC (n = 45 pairs) cohorts were downloaded as binary alignment map (BAM) files from the TCGA data repository (The Cancer Genome Atlas Research Network et al., 2013; National Cancer institute, 2019), and processed to extract small RNA expression profiles. The FL cohort consisted of small RNA-seq data for 25 human fetal lung samples, obtained as described in (Cohn et al., 2021) and available in the National Center for Biotechnology Information Gene Expression Omnibus (GEO) repository under accession number GSE175462 (Edgar et al., 2002). The BCCA validation cohort comprised 58 paired LUAD and ANL samples obtained with informed written consent from patients at Vancouver General Hospital, with approval from the University of British Columbia/BC Cancer Agency Research Ethics Board (H15-03060), as previously described (Cohn et al., 2021). Small RNA-seq data for BCCA cohort samples were downloaded from the GEO repository under accession number GSE175462 (Edgar et al., 2002). All cohorts excluded samples with fewer than five million reads from the analysis to ensure data quality and reliability. The clinical characteristics of the patients included in each cohort are summarized in Table 1.

**TABLE 1:** Demographic and clinical characteristics of patients in TCGA LUSC, TCGA LUAD, and BCCA LUAD cohorts.

Characteristics	TCGA-LUSC (n = 45 pairs)	TCGA-LUAD (n = 46 pairs)	BCCA-LUAD (n = 58)
Median Age (range) years	68 (45–85)	67 (47–85)	70 (45–86)
Sex			
Male	32 (71.11%)	20 (43.48%)	12 (20.69%)
Female	13 (28.89%)	26 (56.52%)	36 (62.07%)
Stage			
IA	5 (11.11%)	10 (21.74%)	18 (31.03%)
IB	16 (35.56%)	9 (19.57%)	18 (31.03%)
II	1 (2.22%)	0	0
IIA	7 (15.56%)	8 (17.39%)	2 (3.45%)
IIB	5 (11.11%)	3 (6.52%)	11 (18.97%)
IIIA	3 (6.67%)	3 (6.52%)	4 (6.90%)
IIIB	0	1 (2.17%)	1 (1.72%)
IV	1 (2.22%)	0	1 (1.72%)
N/A	7 (15.56%)	12 (26.09%)	3 (5.2%)

N/A, not available.

### 2.2 Evaluation of piRNA expression

FASTQ files were obtained by converting BAM files using SAMtools (v1.17) (Danecek et al., 2021) for all cohorts. Reads with a mean Phred score below 20 were discarded to ensure high-quality data using Trimmomatic (v. 0.39) (Bolger et al., 2014). The quality-filtered FASTQ files were processed using the online platform miRMaster 2.0 (Fehlmann et al., 2017). Default miRMaster 2.0 settings were applied, with the following modifications: a custom protocol incorporating a 3′ adapter sequence of ATC​TCG​TAT​GCC​GTC​TTC​TGC​TTG​T, a minimum read length of 15, a sliding window requiring a quality threshold of 1, the STAR alignment algorithm, and a minimum read stack height of 5. Normalization was performed using reads per million (RPM). For patients with duplicate or triplicate tumor samples, expression data were averaged. Following miRMaster 2.0 processing, additional manual expression filtering was performed. For each sample group, piRNAs were considered ‘expressed’ if detected at ≥ 1 RPM in at least 10% of samples within each tissue type. piRNAs that did not meet this criterion were classified as ‘undetected’. Figures representing piRNA expression were generated using R packages: ggplot2 (v. 3.5.1) for data visualization (Wickham, 2016), UpSetR (v. 1.4.0) for intersection analysis (Conway et al., 2017), and pheatmap (v. 1.0.12) (Kolde, 2019). Hierarchical clustering was performed using Euclidean distance (clustering_distance_cols = “euclidean”) to measure dissimilarities between samples and Ward’s method (clustering_method = “ward.D”) for linkage. The resulting dendrogram was used for heatmap representation.

### 2.3 Differential expression and classification of oncofetal piRNAs

Differential expression was assessed using the Wilcoxon test for each piRNA between two sample groups: tumor vs. ANL and FL vs. ANL. We performed a Wilcoxon test for paired samples for tumor vs. ANL comparisons, whereas, for FL vs. ANL, we used the test for unpaired samples. All statistical analyses were conducted in R Studio (v. 4.3.1) (RStudio Team, 2020) using the wilcox. test function from the stats package (v. 4.4.1) part of R (R Core Team, 2013). The fold change (FC) was calculated by dividing the mean expression of the first group by that of the second group. The p-values were adjusted using the Benjamini-Hochberg (BH) correction to control the false discovery rate (Benjamini and Hochberg, 1995). piRNAs were considered differentially expressed if they met the following criteria: an adjusted p-value <0.05 and FC > 2. A piRNA was classified as oncofetal if: (a) it was undetected in ANL samples, meaning it did not meet our predefined criteria in ANL (as detailed in section 2.2), and (b) it was overexpressed in both tumor and fetal lung samples compared to ANL (BH p < 0.05 and FC > 2).

### 2.4 Construction and validation of oncofetal piRNA prognostic model

Survival analyses were conducted using R Studio (v. 4.3.1) (RStudio Team, 2020) to investigate the association between oncofetal piRNA expression and patient outcomes. All tumor samples from TCGA-LUAD (n = 378) and TCGA-LUSC (n = 470) with available survival information were included in this analysis. Small RNA-seq data were downloaded and processed as described in Sections 2.1 and 2.2. Clinical and survival data for these cohorts were retrieved from the University of California Santa Cruz (UCSC) Xena Browser (Goldman et al., 2020). Survival analysis was also performed on the BCCA-LUAD cohort (n = 58). To assess the prognostic relevance of individual piRNAs, we conducted a univariate Cox proportional hazards regression analysis using the coxph () function from the survival package in R (v. 3.6-4) (Therneau and Grambsch, 2000; Therneau, 2024). The dependent variable was overall survival (OS), defined by survival status and time, while the independent variable was the expression level of each piRNA. The hazard ratio (HR) and p-values were extracted from the Cox model summary, and piRNAs with p < 0.05 were considered significantly associated with OS. Following the univariate analysis, a multivariate Cox proportional hazards regression model was constructed to evaluate the combined prognostic value of significant piRNAs. The coxph () function was used, incorporating significant piRNAs as covariates. This model estimated the combined effect of all selected piRNAs on survival. A risk score was calculated for each patient based on the regression coefficients of the multivariate Cox model and the expression levels of the piRNAs. The formula for the risk score is the sum of the product of each piRNA’s expression level and its corresponding regression coefficient. These risk scores were used to stratify patients into two groups (high and low risk) based on the median risk score. The survival probability of the two groups (high vs. low risk) was compared using Kaplan-Meier survival curves. The survfit () function from the survival package was used to fit the survival curves, and the log-rank test was performed to assess the statistical significance between groups.

### 2.5 Evaluation of prognostic performance

To evaluate the prognostic accuracy of the risk score, a receiver operating characteristic (ROC) curve was generated using the survivalROC package (v. 1.0.3.1) (Heagerty and Saha-Chaudhuri, 2022). The time-dependent ROC analysis was performed at the median OS time. The area under the curve (AUC) was calculated to assess the model’s discriminative ability, where AUC = 0.5 indicates no predictive power and AUC = 1.0 indicates perfect discrimination. The concordance index (C-index) was calculated using the concordance. index () function from the survcomp package (v. 1.54.0) (Haibe-Kains et al., 2008; Schröder et al., 2011) to assess the model’s ability to rank patients based on risk scores correctly. The C-index ranges from 0.5 (random prediction) to 1.0 (perfect prediction). To ensure the robustness and stability of the C-index, bootstrap resampling (1,000 iterations) was performed (Efron, 1982). In each iteration, a new dataset was generated by randomly sampling (with replacement) from the original cohort, and the C-index was recalculated. The distribution of bootstrap-derived C-index values was visualized using a density plot, providing insight into variability and confidence in the model’s performance.

## 3 Results

### 3.1 Comprehensive piRNA expression profiling of human fetal lung tissue, LUSC, and LUAD

**FIGURE 1:**
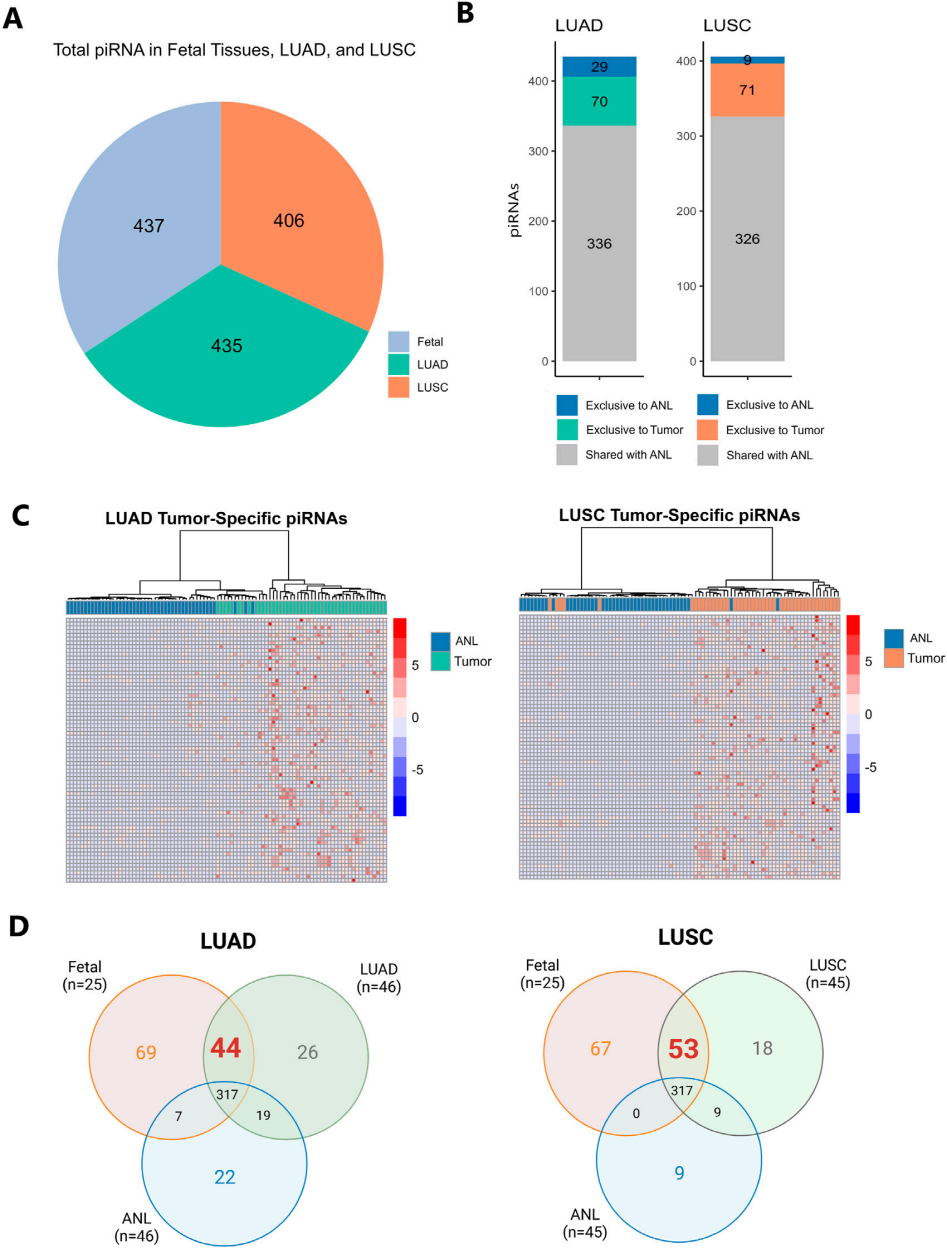
**(A)**. Pie chart depicting the total number of piRNAs identified in lung adenocarcinoma (LUAD), lung squamous cell carcinoma (LUSC), and fetal lung samples. piRNAs were considered “expressed” if they were detected at ≥1 reads per million (RPM) in at least 10% of samples within each tissue type. **(B)** Stacked bar graph showing the number of piRNAs expressed in LUAD and LUSC, highlighting those exclusive to tumor samples, those exclusive to ANL samples, and those expressed in both tissues. **(C)** Heatmaps with hierarchical clustering showing the expression of piRNAs exclusive to tumor in each tumor type (LUAD and LUSC). The color scale ranges from blue (low expression) to red (high expression) based on RPM values. **(D)** Venn diagrams showing the piRNAs exclusive and shared for tumor, fetal, and ANL samples.

To investigate piRNAs potentially involved in both lung development and tumorigenesis, we characterized piRNA expression profiles across human fetal lung tissue, LUAD, LUSC, and matched ANL tissues. piRNAs were considered expressed if they had ≥1 RPM in at least 10% of samples within each tissue type (fetal, ANL, and tumor). A total of 437 piRNAs were expressed in fetal lung tissue, 435 in LUAD, and 406 in LUSC (Figure 1A; Supplementary Tables S1–S3). Among the piRNAs identified as expressed, 70 (16.1%) in LUAD and 71 (17.5%) in LUSC were exclusively expressed in tumor samples. Conversely, 29 piRNAs (6.67%) in LUAD and 9 piRNAs (2.22%) in LUSC were uniquely expressed in ANL tissues (Figure 1B). Hierarchical clustering analysis revealed distinct piRNA expression patterns that stratified samples into two clusters corresponding to tumor and ANL types, as demonstrated in the LUAD and LUSC heatmaps (Figure 1C). Further analysis of the tumor-exclusive piRNAs revealed that 31 piRNAs were shared between LUAD and LUSC, while 39 piRNAs were specific to LUAD and 40 were unique to LUSC (Supplementary Table S4). This suggests that some piRNAs may be involved in subtype-specific aspects of cancer biology. In contrast, shared piRNAs between LUAD and LUSC may play critical roles in maintaining essential characteristics of lung cancer. For each subtype of lung cancer, we identified specific piRNAs that were expressed in fetal lung tissue but undetected in ANL tissue (indicating they did not meet our expression criteria as defined in the methodology). We found 44 piRNAs in LUAD and 53 in LUSC expressed in both tumor and fetal samples, but undetected in ANL. These piRNAs, exhibiting expression patterns reminiscent of fetal lung, could serve as potential candidates for oncofetal reactivation (Figure 1D). For more details, consult Supplementary Tables S5, 6.

### 3.2 Oncofetal piRNAs are reactivated in cancer

**FIGURE 2:**
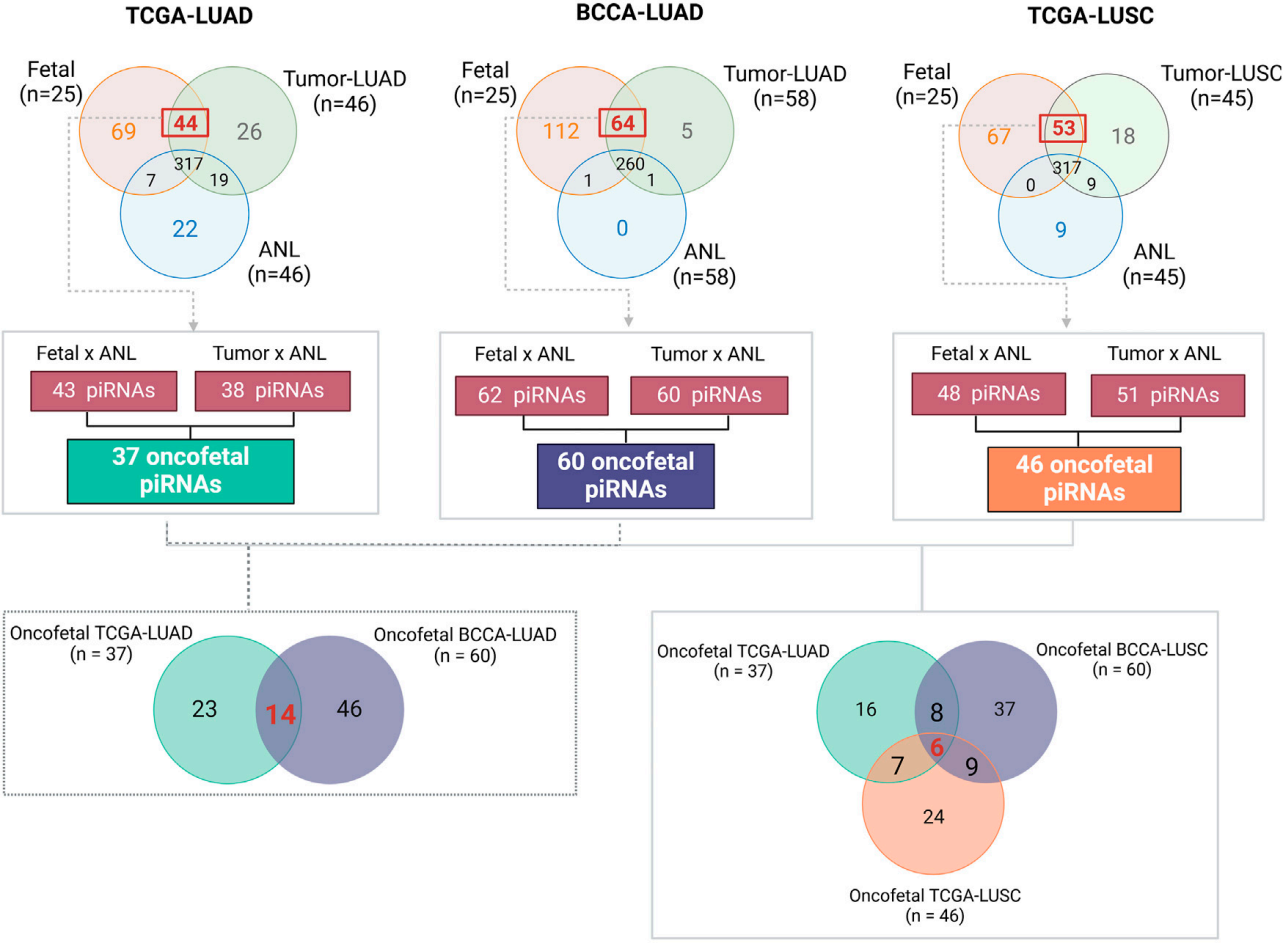
This figure summarizes the methodology and key findings in identifying oncofetal piRNAs. First, for each cohort, only piRNAs in ANL samples (i.e., those that did not meet our predefined expression criteria, as detailed in the methodology) were selected for differential expression analysis using the Wilcoxon test. Next, we identified piRNAs overexpressed in both fetal lung and tumor samples within each cohort. Only piRNAs consistently overexpressed in both groups were classified as oncofetal piRNAs. The Venn diagram on the left highlights the 14 oncofetal piRNAs validated in the BCCA-LUAD cohort for lung adenocarcinoma, while the Venn diagram on the right displays the six oncofetal piRNAs shared across all three cohorts. BCCA, British Columbia Cancer Agency; LUAD, Lung Adenocarcinoma; LUSC, Lung Squamous Cell Carcinoma; TCGA, The Cancer Genome Atlas.

To identify oncofetal piRNAs, we compared the piRNA expression profiles of human fetal lung samples with those of LUAD, LUSC, and patient-matched non-malignant lung tissue (ANL) (Supplementary Figure S1). We identified 43 piRNAs overexpressed in fetal lung compared to ANL and 38 in tumor compared to ANL (Supplementary Table S7). Of these, 37 were overexpressed in both fetal and tumor samples and were classified as oncofetal piRNAs (Supplementary Table S8). Validation in the BCCA-LUAD cohort confirmed 14 of these 37 oncofetal piRNAs (Supplementary Table S9). In the TCGA-LUSC cohort, 48 piRNAs were overexpressed in fetal lung compared to ANL and 51 in tumor compared to ANL (Supplementary Table S10), leading to the identification of 46 oncofetal piRNAs (Supplementary Table S11). Six oncofetal piRNAs were consistently identified across all three datasets (LUAD, LUSC, and fetal lung), including piR-58186, piR-50725, piR-50446, piR-35175, piR-35174, and piR-30926. Figure 2 provides a detailed methodological overview and summarizes our findings across all datasets. In Supplementary Figure S2, we present heatmaps to illustrate the distinct expression patterns of oncofetal piRNAs across each cohort.

### 3.3 Oncofetal piRNA prognostic signature predicts high-risk patients in lung adenocarcinoma

**FIGURE 3:**
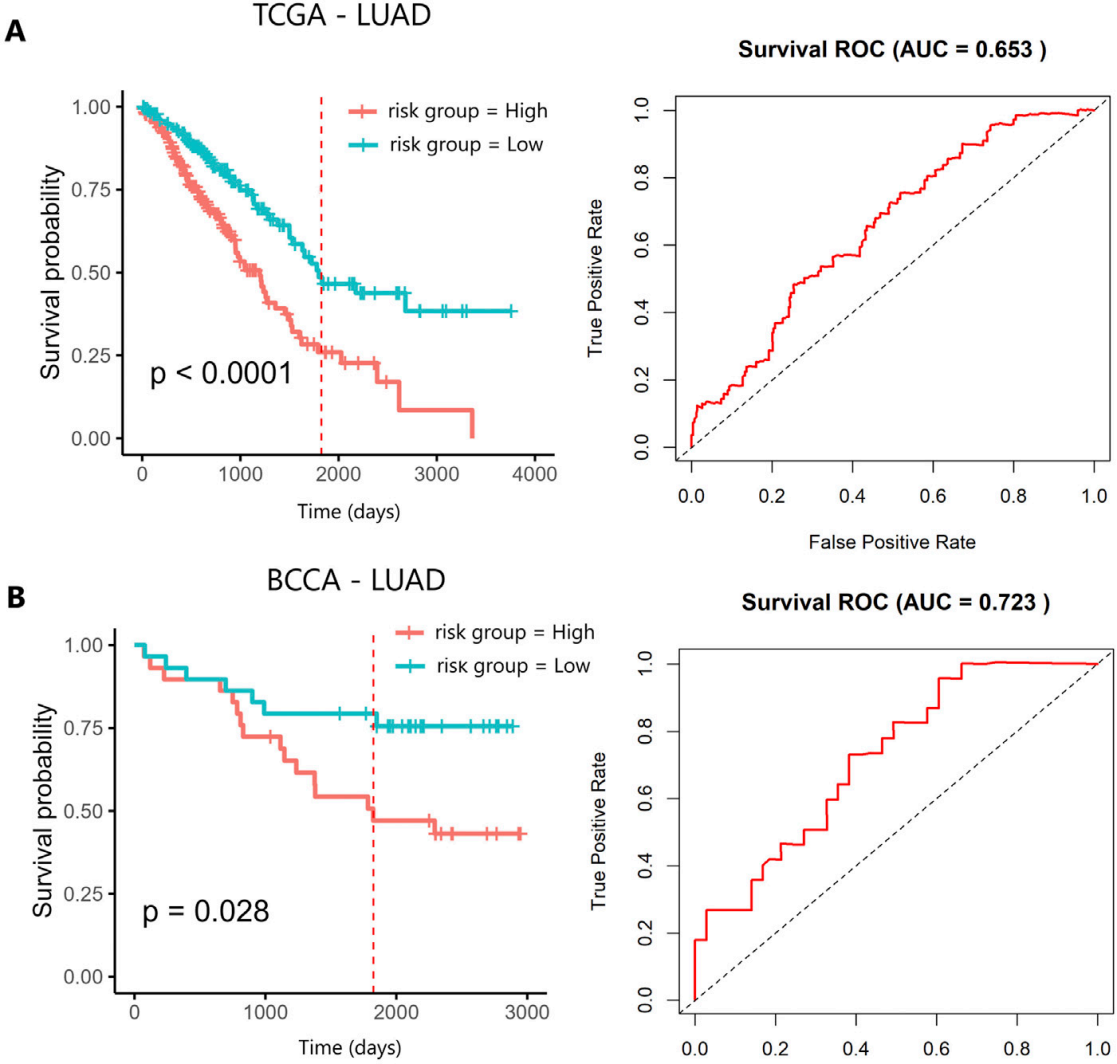
Kaplan–Meier survival analysis of the 8-oncofetal piRNA prognostic signature in **(A)** TCGA-LUAD and **(B)** BCCA-LUAD. The red vertical line marks the five-year survival threshold. AUC, Area Under the Curve; BCCA, British Columbia Cancer Agency; LUAD, Lung Adenocarcinoma; TCGA, The Cancer Genome Atlas.

Oncofetal piRNAs identified in TCGA-LUAD and TCGA-LUSC were used to construct a prognostic model for each histological subtype. For TCGA-LUAD, univariate Cox regression analysis was performed using 37 oncofetal piRNAs, of which eight showed prognostic significance (p < 0.05): piR-41794, piR-44716, piR-44715, piR-33687, piR-34804, piR-33686, piR-33519, and piR-61135 (Supplementary Table S12). Kaplan-Meier (KM) survival analysis stratified patients into high- and low-expression groups based on the median expression level of each oncofetal piRNA to assess survival differences. Among the eight prognostic oncofetal piRNAs, three (piR-33687, piR-33686, and piR-61135) were significantly associated with OS when patients were categorized by high and low expression (Supplementary Figure S3). This discrepancy may arise because Cox regression considers continuous expression values and accounts for time-to-event data, increasing sensitivity to prognostic associations. In contrast, KM analysis relies on dichotomized expression groups, which can reduce statistical power. To evaluate the combined prognostic value of these eight oncofetal piRNAs identified in the univariate analysis, a multivariate Cox regression analysis was conducted, and a risk score was established as described in the methodology (Supplementary Table S13). Patients were divided into high- and low-risk groups based on median risk score. KM analysis of the oncofetal-piRNA prognostic signature showed a significant difference in OS between high- and low-risk groups (p < 0.0001) (Figure 3A). The AUC of the ROC curve was 0.65, demonstrating moderate predictive performance (Figure 3A). The prognostic model was further evaluated using the concordance index (C-index), which measures the agreement between actual OS and model predictions (where a C-index of 0.5 indicates no prognostic value and values closer to 1 indicate better predictive accuracy). The C-index for TCGA-LUAD was 0.63, suggesting moderate predictive accuracy (Supplementary Figure S4A). To validate the model, we applied the oncofetal piRNA prognostic signature to the BCCA cohort. The signature successfully stratified patients into high- and low-risk groups (p < 0.05) (Figure 3B). In BCCA-LUAD, the AUC for survival prediction was 0.72, indicating better predictive performance compared to TCGA-LUAD (Figure 3B). The C-index in the validation cohort was 0.65 (Supplementary Figure S4B). When stratifying by tumour stage, the prognostic signature performed significantly in both early and advanced stage tumors with marked separation in early-stage cancers (Supplementary Figure S5). Furthermore, the piRNA-based model performed comparably to the Tumour, Node, Metastasis (TNM) system, which is a gold standard clinical prognostic marker for NSCLC (Supplementary Figure S6) (Zens et al., 2021).

For TCGA-LUSC, univariate Cox regression analysis identified five prognostic oncofetal piRNAs (p < 0.05): piR-34789, piR-55152, piR-55151, piR-34342, and piR-33404 (Supplementary Table S12). KM survival analysis stratified patients into high- and low-expression groups based on the median expression level of each oncofetal piRNA, and found two of these piRNAs (piR-55151 and piR-55152) were significantly associated with OS (p < 0.05) (Supplementary Figure S7). However, when evaluating the combined prognostic signature in LUSC, the model could not stratify patients into high- and low-risk groups (p > 0.05), indicating that the signature did not have prognostic value in this cohort.

## 4 Discussion

Among sncRNAs, piRNAs have garnered interest due to their abundance, evolutionary conservation, and known roles in gene regulation and development (Mei et al., 2013). However, their functional significance in cancer biology, particularly in lung cancer, remains limited. There have been a handful of piRNAs studied for their association with lung cancers. For example, piR-651, piR-52200, and piR-34971 have been shown to be upregulated in NSCLC, with piR-651 linked to cancer progression (Li et al., 2016), potentially through regulation of cell cycle control proteins such as cyclin D1 and CDK4 (Li et al., 2016). Other piRNAs, including piR-55490, piR-46545, and piR-35127, have been downregulated in lung cancer and demonstrated tumor growth suppression by modulating the AKT/mTOR pathway (Peng et al., 2016; Reeves et al., 2017). Alterations in PIWI proteins, which are central to piRNA biogenesis through slicer activity, may contribute to the deregulated expression of piRNAs in cancer. Changes in expression, such as overexpression (*e.g.*, PIWIL1) or downregulation (*e.g.*, PIWIL2), has been identified in several cancers and are associated with poor prognosis (Navarro et al., 2015; Zhang et al., 2023). Possible mechanisms include disruption of piRNA amplification, aberrant transcriptional silencing of tumor suppressor genes (Zhang et al., 2023), as well as piRNA-independent oncogenic function (Shi et al., 2020; Zhang et al., 2023).

Although piRNAs are implicated in germline tissue maintenance and somatic tissues within various cancers, our knowledge on their presence in fetal tissues is limited and the overlap between these two domains has not been previously explored until this study. piRNAs are expressed in embryos and fetal germline tissues (Roovers et al., 2015) and the placenta (Martinez et al., 2021a; 2021b), yet their role in somatic fetal tissues, particularly lung tissue, has been understudied. The expression of PIWI pathway proteins, including VASA and PIWIL2, is not prominent in first-trimester embryos but becomes evident in second-trimester fetal ovaries (Roovers et al., 2015). This observation aligns with our findings of a strong piRNA population in second-trimester fetal lung tissue, supporting the relevance of piRNAs in both developmental and cancer contexts.

In adult lung tissues, we identified 435 piRNAs in LUAD and 406 piRNAs in LUSC, with the piRNA populations in these tissues showing distinct expression patterns. Previous studies have reported piRNA populations in various cancers, including liver, colorectal, glioblastoma, and gastric cancers (Bartos et al., 2021; Riquelme et al., 2021). While several piRNAs were unique to each lung cancer subtype, 31 piRNAs were shared between them. These shared piRNAs may be involved in maintaining general characteristics in lung cancer such as genomic instability, epigenetic alterations, and immune evasion (Herbst et al., 2018; Hendriks et al., 2024).

A key finding of this study is the identification of 37 piRNAs in LUAD and 46 piRNAs in LUSC that exhibit oncofetal expression patterns. Oncofetal reprogramming can contribute to characteristics shared between tumorigenesis and fetal development including rapid cell division, cellular invasion, migration, and angiogenesis (Ma et al., 2010). Pathways necessary in development such as Wnt, Notch, Hedgehog or TGF-*β* signaling, are commonly affected (Cao et al., 2023). The targets of the oncofetal miRNAs we previously discovered in the LUAD cohort were enriched for Wnt and Hedgehog pathways (Cohn et al., 2021). Similarities in their function to miRNAs and previous identification in regulation of pathways such as Wnt/*β*-catenin may indicate the possibility that piRNAs are linked to development-associated pathways (Deng et al., 2024). Although we did not investigate target prediction due to lack of software availability, we found several oncofetal piRNAs correlated with OS, with a signature of eight piRNAs in the TCGA-LUAD cohort showing prognostic value. These piRNAs were used to generate a risk score that could stratify patients into high- and low-risk groups, with worse OS observed in the high-risk group. This signature was validated in the BCCA-LUAD cohort, showing significant stratification of patients into high- and low-risk groups. The validation results were statistically significant, despite the smaller sample size in the BCCA cohort (n = 58 vs. n = 378 in TCGA). The relative scarcity of deaths in the BCCA cohort, with most samples coming from early-stage lung cancer, did not prevent the signature from demonstrating prognostic potential. These findings suggest that the piRNA signature is a promising predictor of patient outcomes, and further validation in larger cohorts is warranted. In contrast, the LUSC prognostic model failed validation likely due to the cancer’s high molecular and immune heterogeneity, distinct cell-of-origin, fewer well-defined driver mutations than LUAD, and limited availability of robust biomarkers. Together, these factors reduce model generalizability and reproducibility (Lu et al., 2020; Shen et al., 2025).

It is worth noting that piRNA IDs that are consecutive appear to be similar sequences. For example, piR-55152 (chr 8:56,073,866-56,073,905) and piR-55151 (chr 8:56,073,867-56,073,905) which were identified as significant in the TCGA LUSC univariate Cox analysis, and were both associated with OS individually, map to the same locations and have the same sequence other than piR-55152, which is one nucleotide longer than piR-55151. In this study we left them as separate IDs to match the deposition in the RNACentral database. However, functional validation via methods such as qRT-PCR, piRNA mimic or inhibitor studies, luciferase reporter assays, and localization staining, may provide more clarity on whether they are distinct entities or redundant naming. Such approaches offer orthogonal evidence supporting their biological relevance beyond potential technical or computational biases such as algorithmic assumptions, batch effects, reference database constraints, and alignment challenges (Fehlmann et al., 2021).

Although sparse in the literature, some oncofetal piRNAs we identified have been deposited in piRBase, the largest piRNA database, with evidence of their expression in lung cell lines such as H522 cells (a LUAD cell line), and in human bronchial epithelial (HBE) cells (Mei et al., 2015; Wang et al., 2022). Among the piRNAs identified in the TCGA-LUAD cohort, piR-34804 (piR-hsa-26940) and piR-61135 (piR-hsa-25313) were detected in the H522 cell line, with piR-34804 meeting the author’s expression cut-off of 20 reads or more. In the LUSC cohort, piR-33404 (piR-hsa-23555) and piR-34342 (piR-hsa-26492) also met the expression cut-off defined by the authors in H522 cells. piR-34342 exhibited particularly high read counts in H522 cells (227 reads) and was expressed in several other NSCLC and HBE cell lines. Although these piRNAs were not central to the authors findings, evidence of their presence in cell lines warrants further studies to determine their functional roles and significance in lung biology.

## 5 Conclusion

Our study provides novel insights into the role of piRNAs in lung cancer, particularly oncofetal piRNAs uniquely expressed in tumor tissues and associated with patient survival. These findings suggest that piRNAs may serve as potential biomarkers for lung cancer prognosis, with the possibility of further elucidating their functional roles in tumorigenesis. Given the similarities between fetal development and cancer, further investigation into piRNA biogenesis and their regulation in tumor tissues may uncover valuable therapeutic targets for lung cancer.

## Data Availability

The data presented in the study were retrieved from previously published sources. The fetal lung and BCCA LUAD data presented in the study were retrieved from the Gene Expression Omnibus (GEO) repository, accession number GSE175462. The TCGA LUAD and LUSC data were retrieved from the Genomic Data Commons (GDC) Portal. The original contributions presented in the study are included in the article and Supplementary Material. Further inquiries can be directed to the corresponding author.
