# Supplementary material for: Identification of oncofetal PIWI-interacting RNAs as potential prognostic biomarkers in non-small cell lung cancer
Source: Front Genet. 2025 Aug 29;16:1611805. doi: 10.3389/fgene.2025.1611805 (PMC12425789; doi:10.3389/fgene.2025.1611805)
Supplement: Supplementary file 1 [file DataSheet1.zip › Supplementary_Figures/Supplementary_Figure_Captions.docx]

***Supplementary Material Captions***

# Supplementary Data

Supplementary Material should be uploaded separately on submission. Please include any supplementary data, figures and/or tables.

Supplementary material is not typeset so please ensure that all information is clearly presented, the appropriate caption is included in the file and not in the manuscript, and that the style conforms to the rest of the article.

# Supplementary Figures and Tables

For more information on Supplementary Material and for details on the different file types accepted, please see [here](https://www.frontiersin.org/guidelines/author-guidelines#supplementary-material).

## Supplementary Figures

**
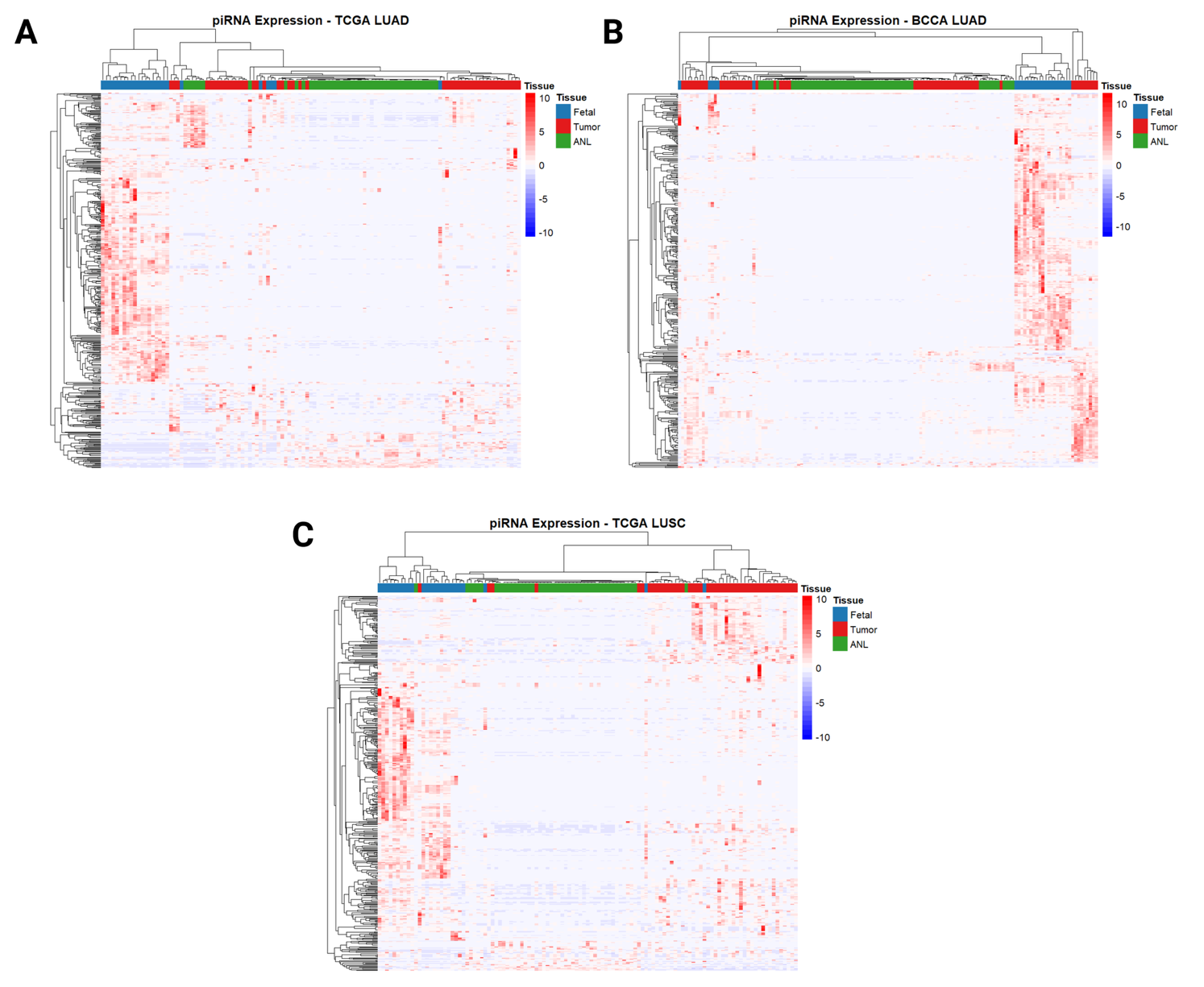
**

**Supplementary Figure 1.** Hierarchical clustering heatmap of piRNA expression across fetal, adjacent normal lung (ANL), and tumor tissues. Expression profiles of all piRNAs detected were included. The heatmap displays row-scaled (z-score) expression values to highlight relative expression patterns. Clustering of both rows (piRNAs) and columns (samples) was performed using Pearson correlation-based distance with average linkage. Sample groups are annotated by color: fetal (blue), ANL (green), and tumor (red).


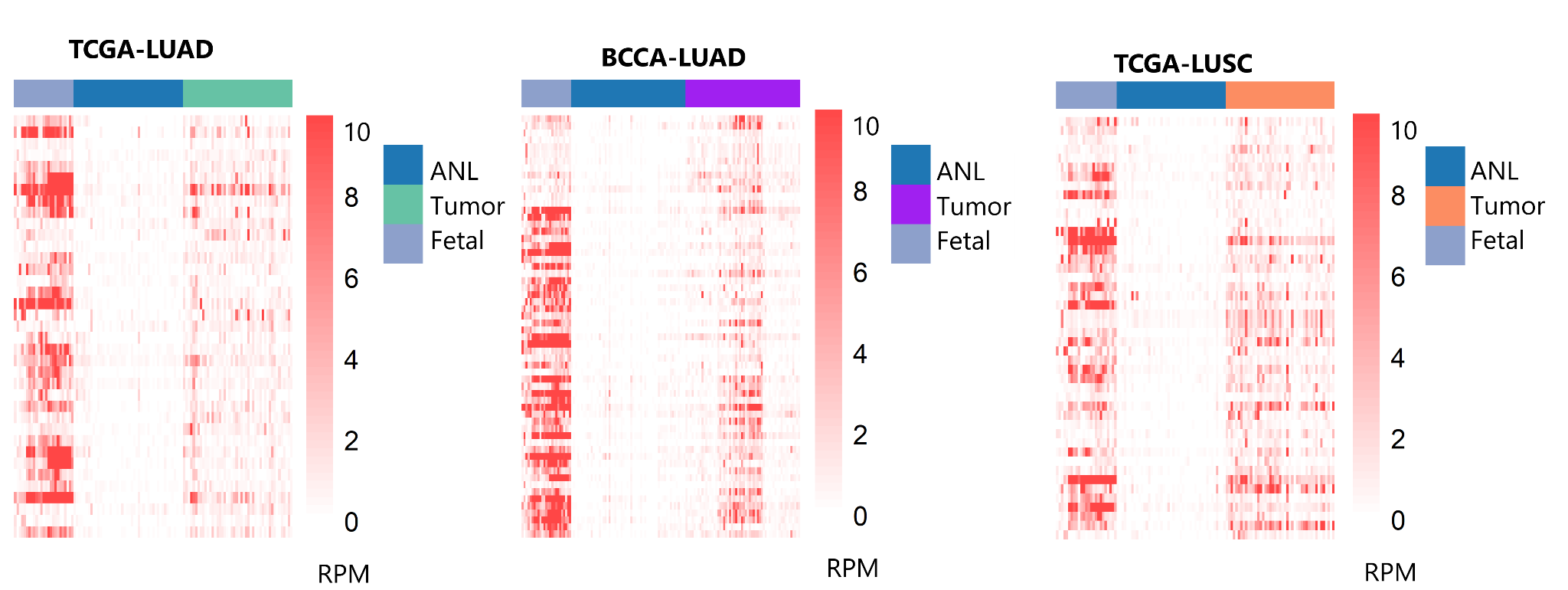


**Supplementary Figure 2.** Heatmap displaying the expression patterns of oncofetal piRNAs identified in each cohort. The color scale ranges from white to red, representing expression levels from 0 to 10 Reads Per Million (RPM). "ANL" refers to patient-matched non-malignant lung tissue.


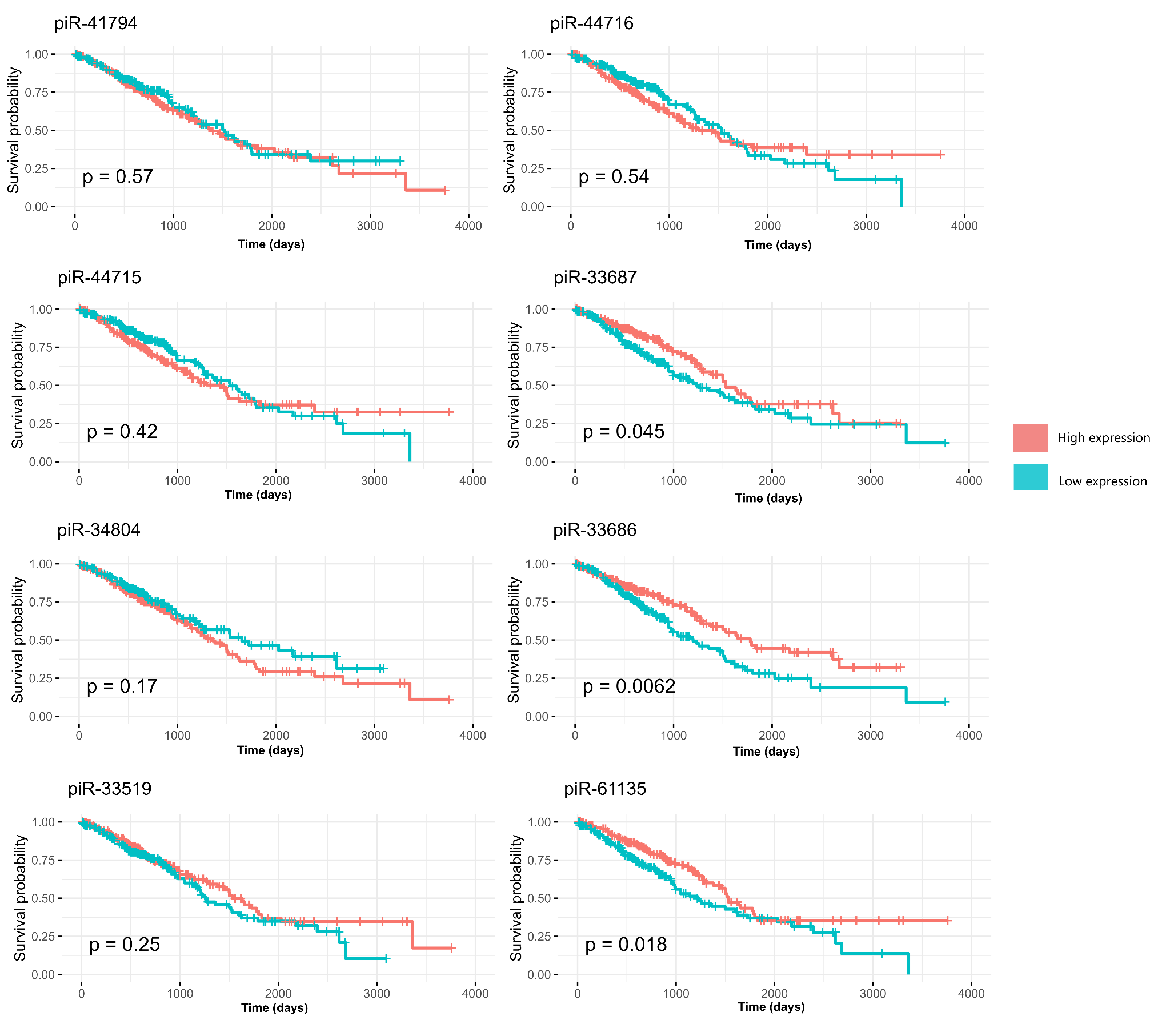


**Supplementary Figure 3.** Kaplan–Meier survival curves of eight oncofetal piRNAs with prognostic significance in TCGA-LUAD cohort. Patients were classified into high- and low-expression groups based on the median piRNA expression levels.


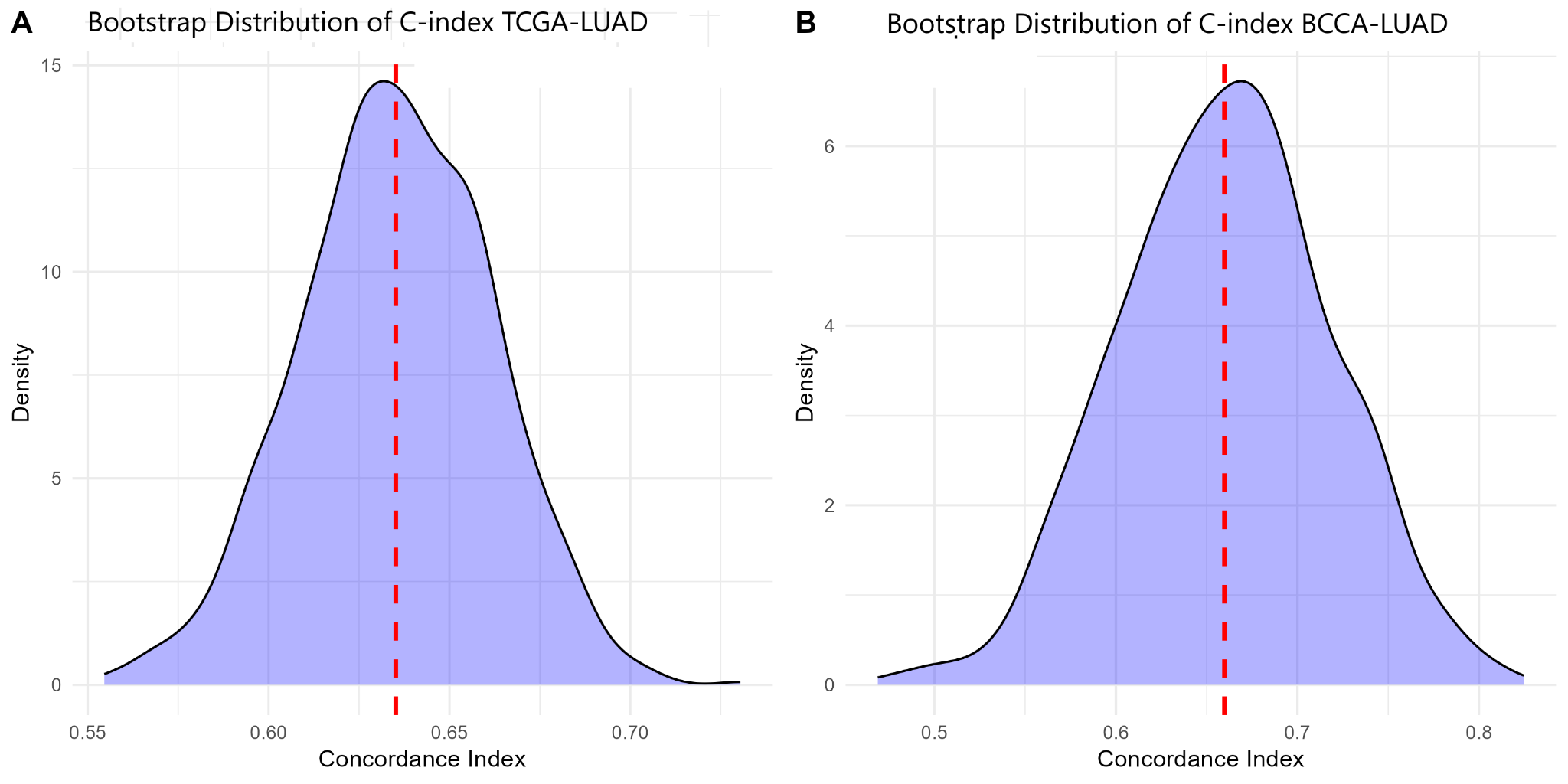


**Supplementary Figure 4.** Distribution of C-indices across bootstrap samples, illustrating the performance of the combined model in **(A)** the TCGA-LUAD cohort and **(B)** the BCCA-LUAD cohort. The red dashed line represents the mean Concordance Index (C-index) from the bootstrapped distribution.

**
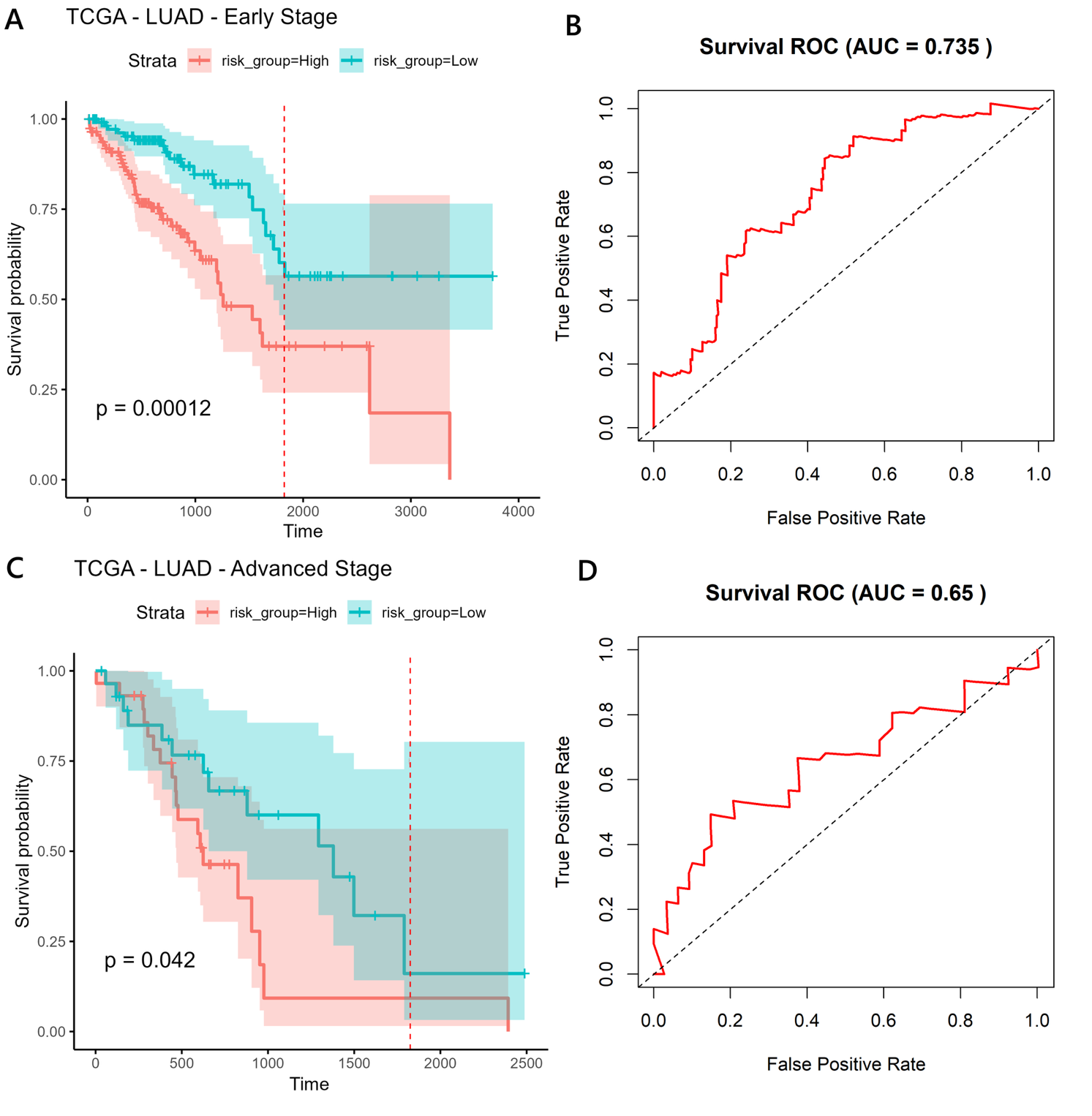
**

**Supplementary Figure 5.** Risk stratification performance in LUAD patients. (A–D) TCGA-LUAD cohort stratified by pathological stage: Kaplan–Meier curves and ROC curves shown separately for early-stage, Stage I, IA, IB, II, IIA, or IIB (A–B), and advanced-stage patients, Stage III, IIIA, IIIB, or IV (C–D). The red vertical line marks the five-year survival threshold. AUC, Area Under the Curve. Samples with missing or undefined stage information were assigned as NA and excluded from subsequent stage-based analyses.

**
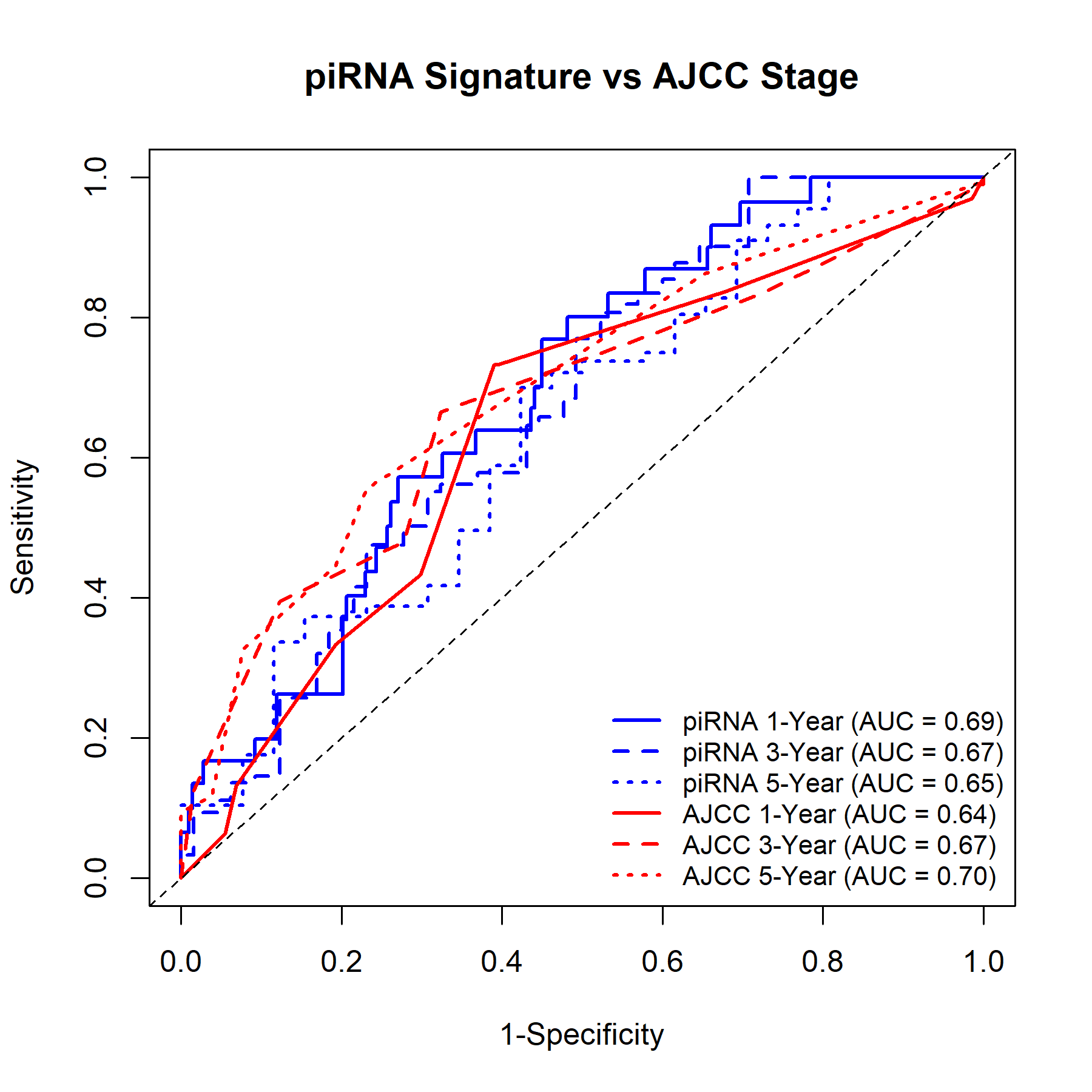
**

**Supplementary Figure 6.** Time-dependent ROC curves comparing the predictive accuracy of the piRNA-based prognostic signature and AJCC pathological stage at 1, 3, and 5 years for overall survival in LUAD (n = 288). Solid, dashed, and dotted lines indicate predictions at 1, 3, and 5 years, respectively.


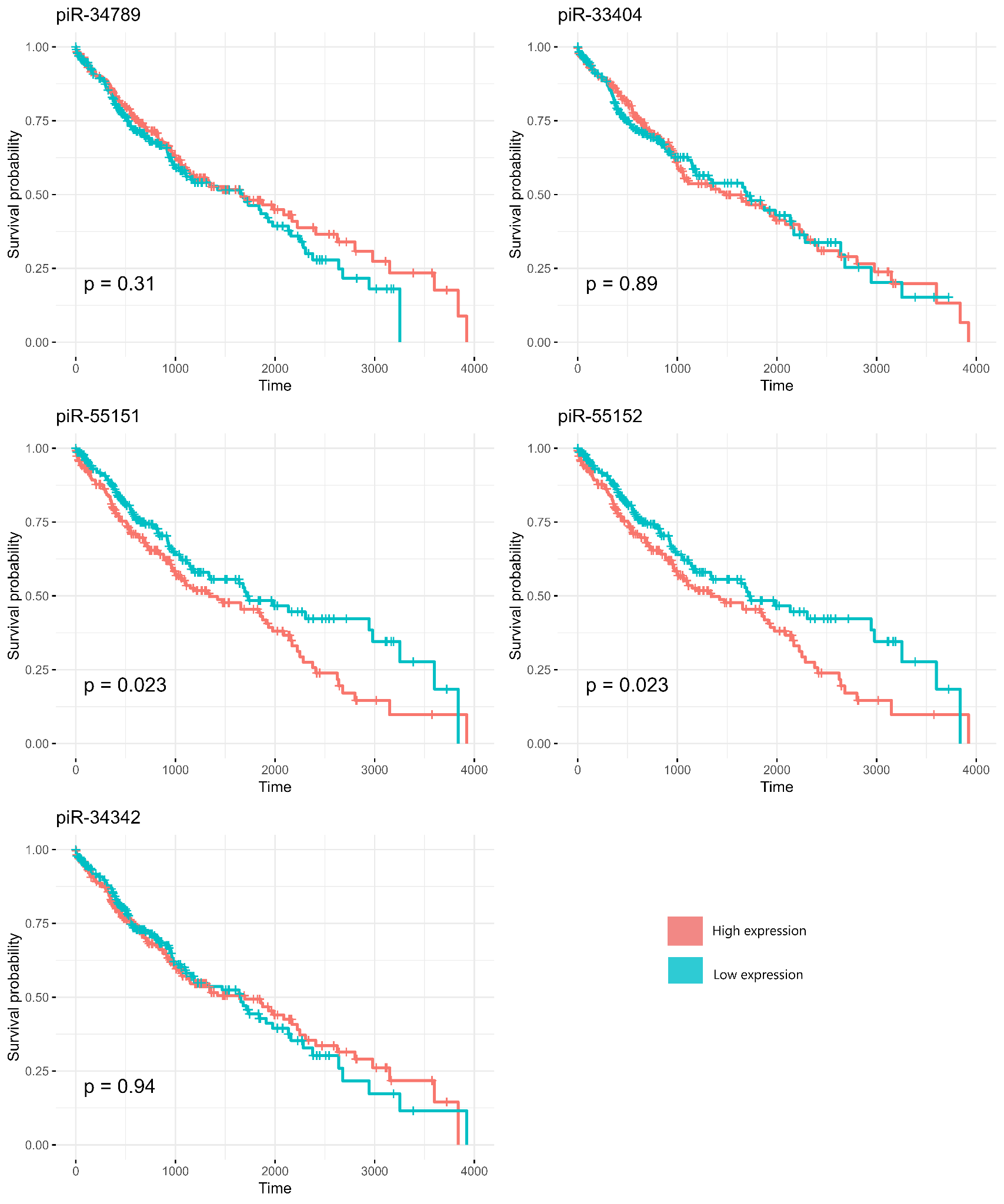


**Supplementary Figure 7.** Kaplan–Meier survival curves of eight oncofetal piRNAs with prognostic significance in TCGA-LUSC cohort. Patients were classified into high- and low-expression groups based on the median piRNA expression levels.
