# Supplementary figures and images for: Identification of oncofetal PIWI-interacting RNAs as potential prognostic biomarkers in non-small cell lung cancer
Source: Front Genet. 2025 Aug 29;16:1611805. doi: 10.3389/fgene.2025.1611805 (PMC12425789; doi:10.3389/fgene.2025.1611805)

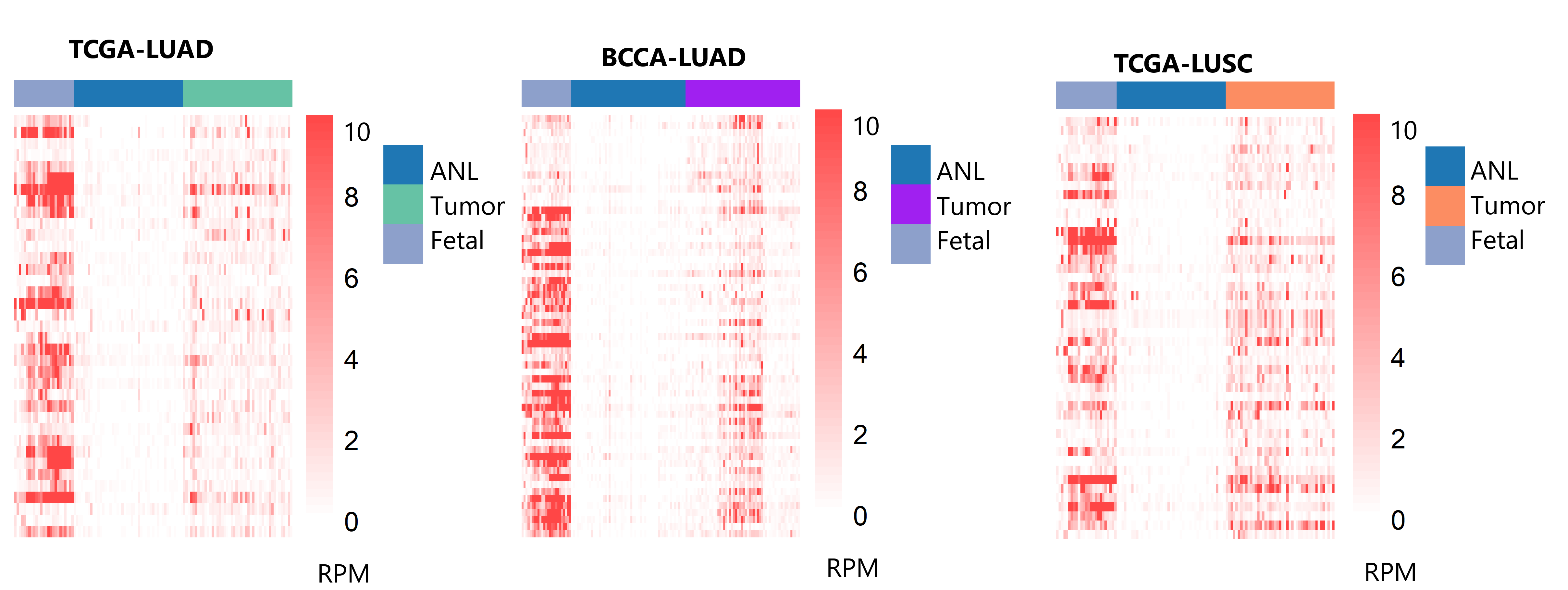

Supplement: Supplementary file 1 [file DataSheet1.zip › Supplementary_Figures/supplementary_figure_2.tiff]

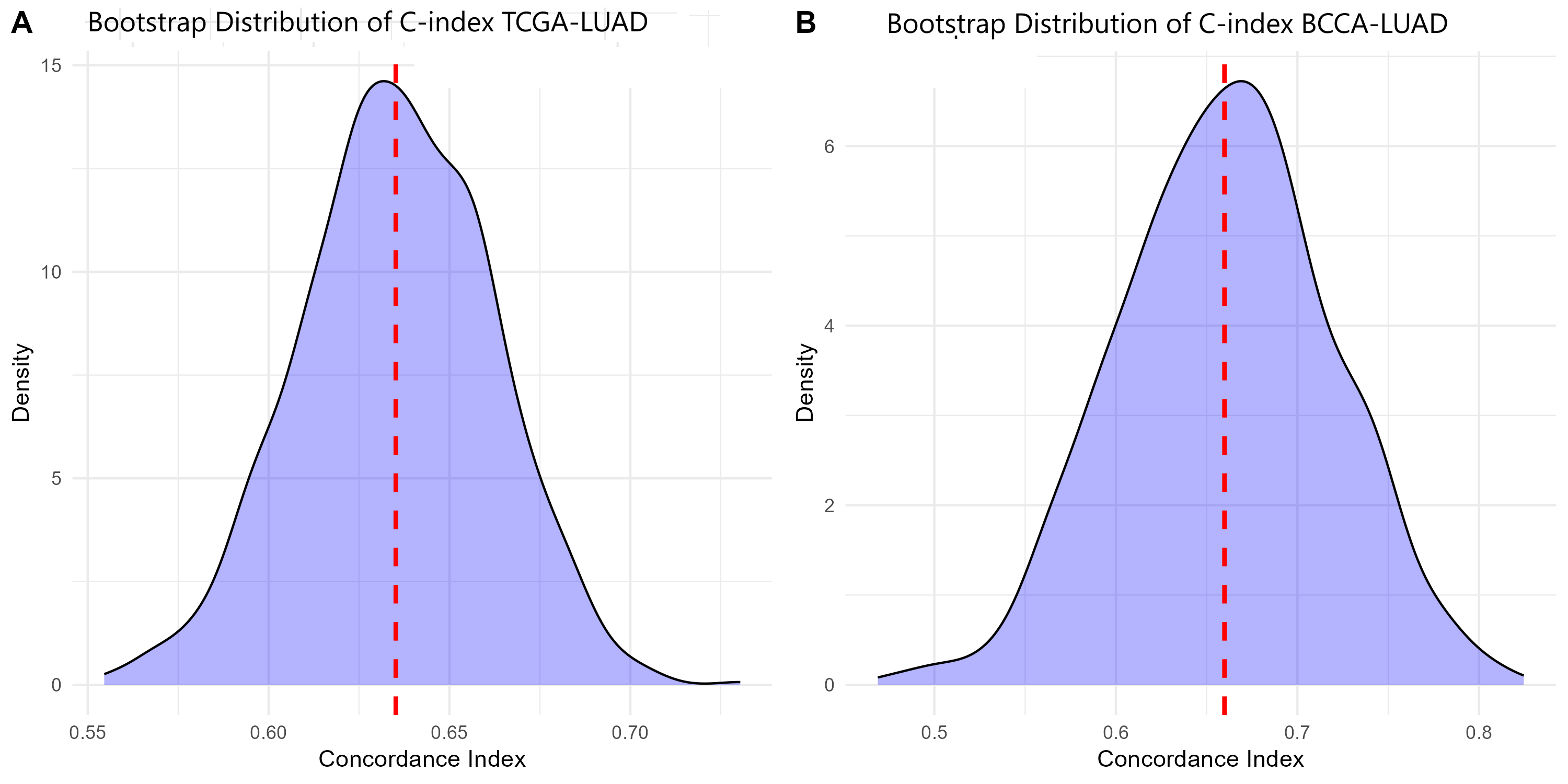

Supplement: Supplementary file 1 [file DataSheet1.zip › Supplementary_Figures/supplementary_figure_4.tiff]

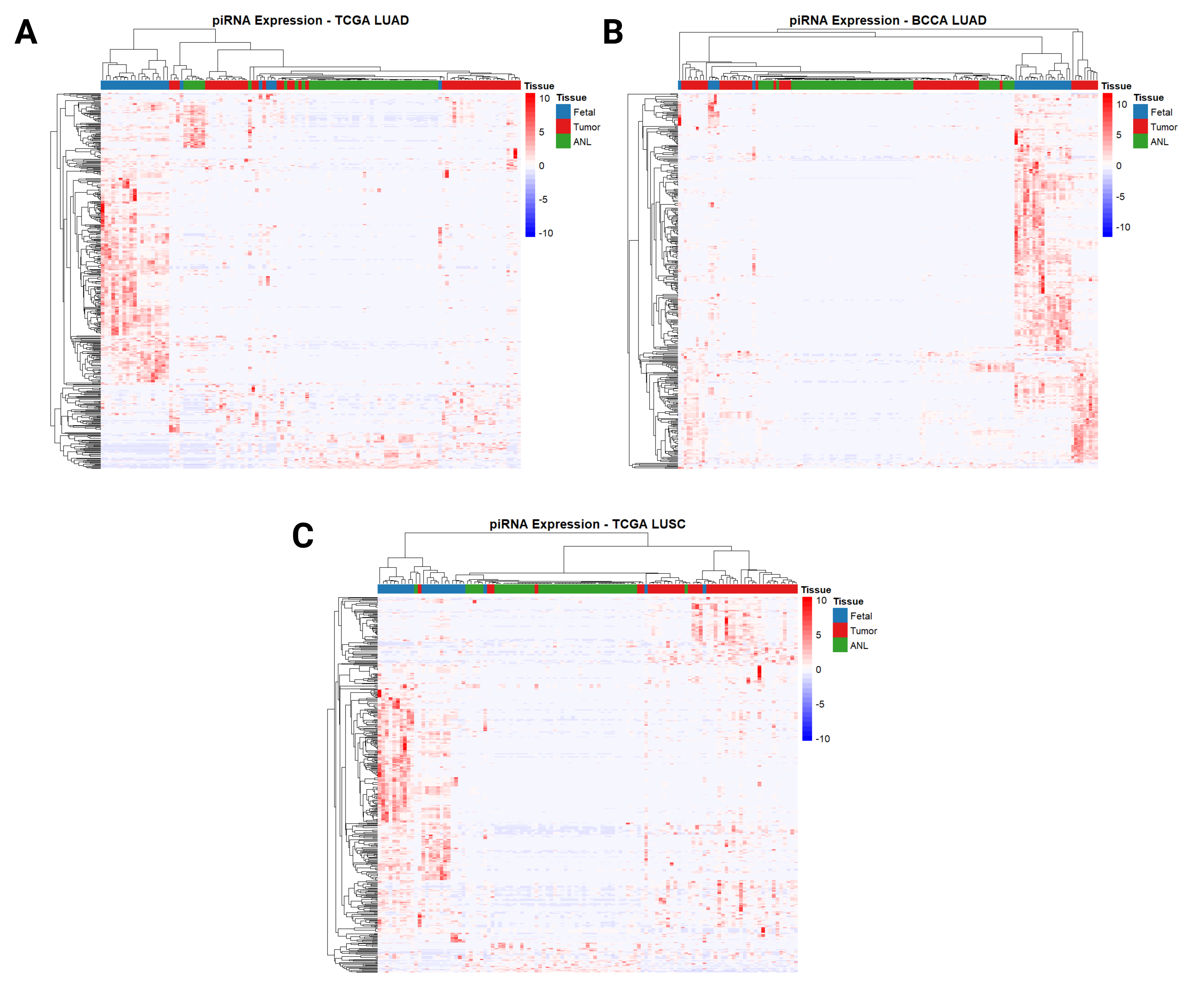

Supplement: Supplementary file 1 [file DataSheet1.zip › Supplementary_Figures/supplementary_figure_1.png]

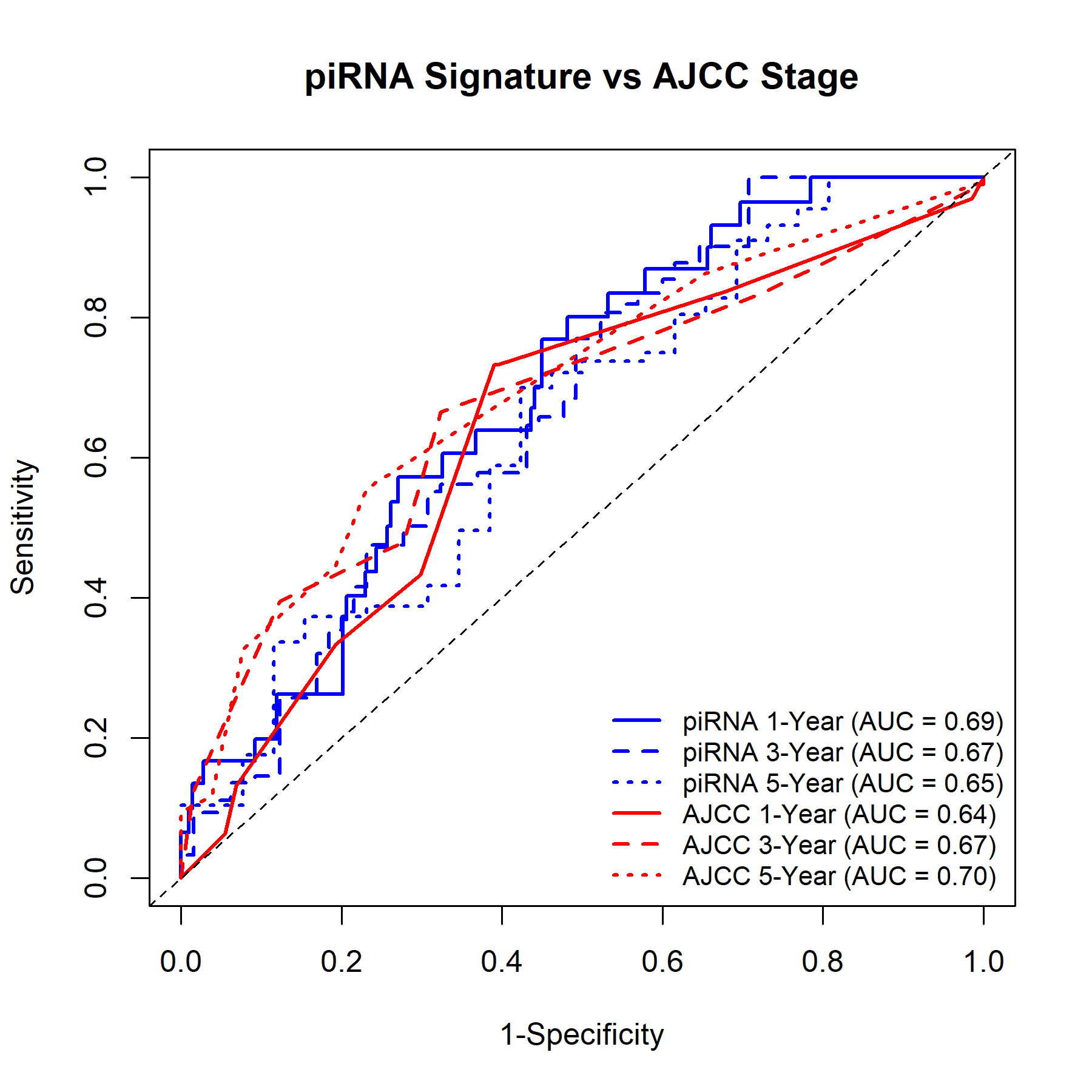

Supplement: Supplementary file 1 [file DataSheet1.zip › Supplementary_Figures/supplementary_figure_6.png]

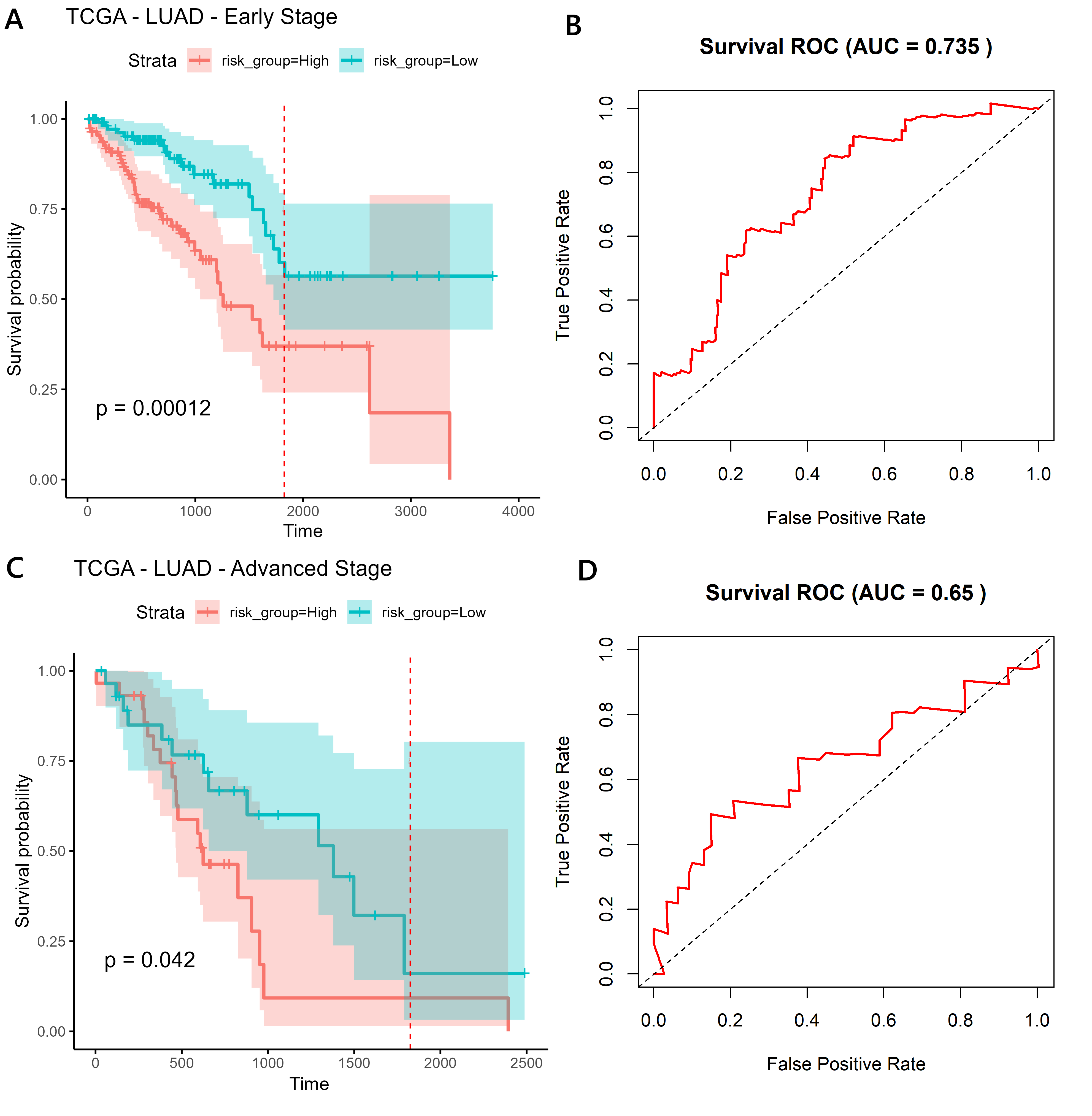

Supplement: Supplementary file 1 [file DataSheet1.zip › Supplementary_Figures/supplementary_figure_5.png]
